# Care Needs and Caregivers: Associations and Effects of Living Arrangements on Caregiving to Older Adults in India

**DOI:** 10.1007/s12126-016-9243-9

**Published:** 2016-04-28

**Authors:** Allen Prabhaker Ugargol, Inge Hutter, K. S. James, Ajay Bailey

**Affiliations:** University of Groningen (RUG), Groningen, The Netherlands; Institute for Social and Economic Change (ISEC), Bangalore, India; Faculty of Spatial Sciences, University of Groningen, Groningen, The Netherlands; Population Research Centre (PRC), Institute for Social and Economic Change (ISEC), Bangalore, India

**Keywords:** Ageing, Caregiving, Care needs, Caregiver, Older adults, Living arrangements, India

## Abstract

As the ageing phenomenon continues in India, we explore the care needs of older adults and identify caregivers for specific care needs across living arrangements. Using the United Nations Population Fund (UNFPA) conducted Building Knowledge Base on Population Ageing in India (BKPAI 2011) data comprising 9850 older adults, we employed statistical methods to analyze the data, find associations and used binary logistic regression to model the adjusted and unadjusted effects of living arrangements on caregiving to older adults for specific care needs. Care-requiring situations considered were acute sickness, sickness requiring hospitalization, chronic morbidity, functional disability represented by ADL and IADL limitations, and locomotor disability. Results indicate that living arrangements of older adults were significantly associated with health, functional status and disability as well as caregiving patterns. Our results suggest that co-residence with children and all others was beneficial to older adults in obtaining care from a family caregiver for their hospitalization and chronic morbidity needs while living with spouse or living with a partner was advantageous for older adults in receiving care for their ADL limitations and during hospitalizations. Mean number of children was also significantly associated with the availability of a caregiver during hospitalization, locomotor disability, chronic morbidity and acute sickness. The study also highlights a little known phenomenon, that there was familial help available to older adults who lived alone. Notably, non-family sources of caregiving were steadily becoming visible (as high as 8–10 % of the caregiving component) especially among older adults living alone.

## Introduction

As India experiences the demographic transition, older individuals are now living longer with increasing life expectancy, and at the same time, are also requiring more assistance or care to manage their day to day activities. As substantially large cohorts are now surviving to older ages in India, there is an increased need for elder care and support. In India, as in most Asian countries, the family is a cherished social institution, and co-residence has been the traditional way by which families met the care needs of their older adults. Studies have reinforced that home-based care with family members as primary caregivers was still the first and often the only option for a majority of older adults, and the most common type of living arrangement in India was found to be living with married sons and their families (Devi and Indira 2007; Prakash 1999). Unfortunately, demographic shifts are reducing the availability of familial support to India’s older adults (Krishnaswamy et al. 2008; Rajan and Kumar 2003). Alongside the increase in population of older adults, there is a simultaneous decline in the number of younger family members available to care for these older adults due to several reasons. There has so far been limited evidence available on older adults’ living arrangements and caregiving patterns for their health and functional needs. Studies on living arrangements of Indian older adults have focussed on their health and functional status for long but have not explored the caregiver type and caregiving patterns for different requirements, limitations and disabilities of older adults (Devi and Indira 2007; Prakash 1999; Sudha et al. 2006; Agarwal 2012). This study aims to explore the care needs of older adults, identify caregivers to older adults for their health and functional needs, and investigate differences in caregiving to older adults’ by their living arrangements. With India’s older adult population currently at 8.57 % of their total population (Census of India 2011), information on living arrangements and caregiving patterns to older adults for their health, functional and disability needs will be pertinent to generate knowledge for debate, policy and action.

In ageing literature, living arrangements have either been defined by household composition or by the number and identity of the cohabitants. Across the world, families have always been considered the mainstay in terms of caregiving to older adults. Even in the United States nearly two-thirds of the 5.5 million older adults with chronic disabilities rely, often exclusively, on family members for help with basic activities of daily living (Spillman and Pezzin 2000). Co-residence has been recognised as an important mode of support that adult children provide for their elderly parents. While a quarter to half of older adults in the developed world might have adult children co-residing, co-residence of older parents and adult children ranges from two-thirds to three-quarters across Asia, Africa and Latin America (United Nations 2005). Family roles involve expectations and obligations that shift over the course of life and living arrangements have been closely connected with co-residence status, living with/without a partner and marital status in much of published literature. There is also evidence of a close relation between health and well-being of older adults and their living arrangements (Sereny 2011; Agarwal 2012); however, there is no information on who dons the caregiving role in such a scenario. In a patriarchal society, where gender roles dominate and where women do most of the housework and caregiving, co-residence with one’s spouse may be more beneficial for men since women handle the caregiving role. Interestingly, Indian women have also benefitted from the presence of the spouse and sons in the household, owing mainly to their general dependence on significant others and the socio-cultural security of having a surviving spouse (Lamb 1999).

A complex familial exchange of care ensues between parents and children and also among the extended family and social networks of the elderly in many Asian cultures including India (Agree et al. 1999). Traditional multi-generational households have historically represented an intricate social network system that ensured care for older adults from within the family or within their extended social network. Unfortunately, traditional family-based care is becoming less common than in the past in India (Arokiasamy et al. 2010) due to the following reasons. Firstly, fertility reductions led to fewer children being available to care for older family members and secondly, with increased education, migration for employment and better economic opportunities, movement of adult children away from the traditional household has become inevitable leaving behind older dependents (Visaria 1999; Deshingkar and Akter 2009). While it becomes imperative to explore how the institutions of family and healthcare adapt and cope with the demographic and health transition that challenge the care of the elderly (Lee 2003; Lloyd-Sherlock 2010), very little evidence exists from India on the form and extent of care received by the elderly in the changing scenario. Increased requirement of care is also not free from financial burden; however, in the Indian context, receiving paid or formal care may not be feasible or culturally appropriate for most of the older adult population. Even with regard to opting for formal care such as in times of medical need, the decision to go in for formal care is taken by family and extended family (Levkoff et al. 1999) who often remain primary caregivers. Very few studies in India have looked at care requirements of older adults and their sources of care and support, however, these have been very localized in nature (Panigrahi 2009; Sudha et al. 2006). Studies thus far on older adults’ health status and caregiving patterns in India have been fragmented and although there is some evidence on the association between older adults’ living arrangement patterns and their health status, there is paucity of information on caregiving patterns to older adults by their living arrangements and for specific care needs.

### Understanding Social Change and Familial Support for Older Adults in India

Historically, India does not have a well-developed social security system since a majority of the workforce belongs to the informal sector. Hence, work related pension is available only to around 10 % of the Indian population who had been part of the formal workforce. In the absence of a formal social support programme in India, older individuals tend to depend on filial piety and intergenerational support during their old age (Gupta and Pillai 2002) for financial as well as other forms of support. A strong tradition of filial piety is visible in India and is expressed through co-residence of older adults with their adult children. This co-residence is known to facilitate support systems between the elderly and their adult children (Chan 1997; Knodel and Chayavon 1997). However, the flow of support is not unidirectional from the adult child to the older parent but rather of a reciprocal nature. Studies have also shown that there are benefits to older adults’ health on account of co-residence, particularly in terms of the relationship between co-residence and self-rated health, chronic and short-term morbidity (Sudha et al. 2006; Agarwal 2012).

Recently, living alone has been on the rise in India. In the absence of a financial safety net for older adults living alone, this adds a new potential poverty dimension to ageing in India (Husain and Ghosh 2011). The NSSO data (60th Round) conducted in 2004 showed that 5.2 % of India’s older adults lived alone and another 12 % of them lived with their spouse exclusively. The main reasons for living alone were reported as widowhood, not having children or children living elsewhere on account of migration. The recent BKPAI Report (2011) finds that 6 % of India’s older adults live alone and 15 % of them live with their spouses exclusively. We see a gradual trend towards increasing numbers of older adults living alone bereft of their family and gender plays a role too. The Situation Analysis of the Elderly in India (2011), highlighted that nearly 50 % of Indian older adults are dependents, often due to widowhood, divorce, or separation, and a majority of these elderly individuals are women (70 %) and 1 out of 10 elderly women lives alone. In the southern State of Tamil Nadu for example, it was observed that as high as 16.2 % of women lived alone (Report on the Status of Elderly in Select States of India 2011).

### Health, Functional and Disability Needs of Older Adults in India

With modernization, older adults increasingly face barriers to good health status and ‘care’ from within the family on account of family nuclearization and increase in dependency (Gupta and Sankar 2002; Rajan and Prasad 2008). Ill health and disability are known to have a significant impact on economic security, level of independence and social interaction of the elderly too (Bloom et al. 2010). In the recent past, the prevalence of chronic disease among the elderly in India has been showing a steady increase (Situation Analysis of the Elderly in India 2011), particularly cardiovascular, metabolic, and degenerative disorders (Ingle and Nath 2008). Chronic morbidity necessitates constant care and support for older adults; however, with declines in family support, it is pertinent to explore how their care needs are being addressed.

Disability is generally considered a good indicator of overall health status among older populations as it is thought to be the result of cumulative damage created by the chronic disease processes that affect humans throughout life and that manifest themselves in older ages (Fried and Guralnik 1997; Pope and Tarlov 1991). Several theoretical measures have been put forth to explain different levels of physical disability (World Health Organization. WHO 1980; World Health Organization WHO 2002). A very large majority of older adults suffer from diminished functional abilities in physical (eating, bathing, dressing, walking, climbing stairs, getting-up from a sitting position, etc.) as well as sensory (hearing and vision) health domains (Alam 2006). Everyday self-maintenance activities (ADL and IADL) are considered prominent indicators of disability which require assistance (Albert 2004). Impairment in everyday activities indicates cognitive and motor deficits to carry out work-a-day routine tasks. Health and well-being of older adults are thus affected by interwoven aspects of their social and physical environment and family support has been found to be an important factor affecting the health and ability of older adults in managing their daily activities (Devi and Murugesan 2006).

## Objectives

The specific objectives of this study are to investigate the association between living arrangements and the health and functional status of older adults in India, to explore differentials by living arrangements on caregiving patterns to older adults for their health and functional needs and to assess the effect of living arrangements on caregiving patterns to older adults. In particular, we aimed to answer the following questions, namely, are there differentials in older adults’ health and functional status by living arrangements?, are there variations in caregiving to older adults’ health and functional needs by their living arrangements?, and what is the effect of living arrangements on caregiving patterns and do other socio-demographic, family support and socio-economic factors mediate the association?

The paper explores the health and functional needs, care gaps and identifies caregiving patterns to older adults by their living arrangements. By care needs, the special situations in which older adults require assistance such as during times of sickness, functional limitations and disability are considered. Care gaps refer to situations where older adults have to self-assist themselves as significant others who could offer them assistance are either not living with them or due to other reasons are unable to assist them. Caregiver types being explored here include: Self, Spouse, Family Caregiver and Non-Family Caregiver. It is hypothesised that older adults living with spouse, children and/or in family settings would obtain informal care from family caregivers in contrast to older adults who live alone, are without a spouse and who live away from their children. The latter group of older adults is hypothesized to have considerably reduced access to family-based informal care and could be depending more on relatives and non-family caregiving sources such as neighbours, friends, extended social support networks including community and religious institutions apart from formal care.

### Research Design

The data for the study were drawn from a cross-sectional survey of older adults in India conducted by the United Nations Population Fund (UNFPA) under a larger study on ‘Building Knowledge Base on Population Ageing in India’ (Report on the Status of Elderly in Select States of India 2011). The sample consisted of 9850 (N) older adult respondents aged 60 years and above from seven states across India which included Odisha, West Bengal, Maharashtra, Himachal Pradesh, Punjab, Tamil Nadu and Kerala. In each of these Indian States, the proportion of older adults was higher than the national average.

### Variables

#### Living Arrangements:

Living arrangement is the predictor variable in this study and is constructed based on the information about household members living in the household. Living arrangement was categorised into: ‘living alone’, ‘living with spouse’ and ‘living with family’. ‘Living alone’ meant that the older adults were living without the spouse and other kin. ‘Living with spouse’ meant the older adults lived exclusively with their spouse and ‘Living with family’ meant that the older adult lives with spouse (if available), children, son-in-law, daughter-in-law and grandchildren as available.

#### Socio-Demographic:

Variables included are gender, age group, place of residence, marital status and children. Marital status was coded into currently married/living together and currently single (included widowed, separated, deserted and divorced individuals). The mean number of children was included.

#### General Health:

Self-rated health is one of the variables under health characteristics. Respondents had been asked to rate their general health status on a scale of excellent, very good, good, fair and poor. Excellent and very good were grouped together. Good and fair were clubbed into the second category and ‘poor’ health was retained as such.

#### Acute Sickness:

Acute Sickness was measured in response to the question ‘were you sick for any time during the last 15 days without requiring hospitalization’. Positive responses were categorised as 1 (any ailment) and negative responses into 2 (no ailment).

#### Hospitalization:

Particulars of medical treatment received as an inpatient during the past 12 months were measured in response to the question ‘Did you have any major health problem during the last 365 days requiring hospitalization? Positive responses were coded into 1 (any hospitalization) and negative responses into 2 (no hospitalization).

#### Chronic Morbidity:

Chronic Morbidity was measured in response to the question ‘has any doctor or nurse ever told you that you have any of the following ailments?’. The list of chronic ailments comprised of 20 chronic diseases (stroke, heart disease, diabetes, cancer, etc.). Positive responses were coded into 1 (any chronic morbidity) and negative responses into 2 (no chronic morbidity).

#### Functional Limitations:

Respondents were asked to self-report whether they had any difficulty with activities of daily living (ADL) and Instrumental Activities of Daily Living (IADL). The ADL measure consisted of 6 tasks, including bathing, dressing, toileting, mobility, continence and feeding. Responses were coded based on the presence or absence of difficulty into ‘any ADL limitations’ and ‘no ADL limitations’. The IADL scale consisted of eight tasks: using the telephone, daily shopping, preparing meals, doing housekeeping, doing laundry, transportation, taking medications and handling personal finances. Similar to ADL limitations, responses for IADL limitations were coded into ‘no IADL limitations’ and ‘any IADL limitations’. The sum score for IADL reflected functional status with lower scores indicating more intact functional abilities. Mean ADL and mean IADL were also computed.

#### Locomotor Disability:

Respondents were asked if they had difficulty with respect to vision, hearing, walking and chewing. Positive responses (those requiring partial and full assistance) were coded as ‘any one disability’ and negative responses into ‘no disability’.

#### Socio-Economic Characteristics:

Socio-economic measures included the wealth index, financial status of older adults in the household and financial dependency. The wealth index, an indicator of economic status of households, is consistent with expenditure and incomes measures (Rutstein 1999). This index was constructed using information on household assets and housing characteristics. The financial status of older adults was measured with the response categories being ‘not dependent’, ‘partially dependent’ and ‘fully dependent). Whether the older adult contributed to the household expenditure was also ascertained and ‘whom the older adult depends on for financial support to meet basic needs’ was recorded with the response options being Spouse, Any Family, Other relatives/friends, NGO, community, etc.

#### Caregiver Characteristics:

Responses of older adults to questions that ranged from assistance provided for functional assistance in ADL and IADL tasks, chronic morbidity management, acute illness, morbidity including hospitalization, and locomotor disability concerns of older adults were included: who provides assistance for ADL tasks (main caregiver)?, who pays for the treatment expenditures in case of chronic morbidity?, who paid for their acute sickness (outpatient) treatment expenses?, who stayed with the older adults to provide care in the hospital? And who provided aids to older adults to overcome their locomotor disability? The study categorised caregivers into – ‘family caregiver’ (son, daughter, daughter-in-law, son-in-law, unmarried children and grandchildren living in the same household), ‘non-family caregiver’ (any relative or friend), ‘spouse’ and ‘no one’. Any ‘relative’ category includes sibling, cousin, aunt, uncle, niece, nephew living in a separate household while the ‘friend’ category includes friends, neighbours, confidants, co-workers, etc. Since responses to chronic morbidity and ADL limitations were for more than one condition, individual counts were added and caregivers by type for each condition/limitation were totaled to arrive at a cumulative caregiving proportion to older adults. In case of locomotor disabilities, the caregivers who helped older adults’ procure ‘aids’ to overcome the respective disability were taken into account.

#### Care Needs:

The need for care among older adults was established using health, functional limitation and disability conditions which necessitated caregiving to older adults such as the presence of an acute morbidity (sickness) event in the past 15 days, presence of chronic morbidity, sickness requiring hospitalization (any type of sickness that ended up in hospitalization included), functional status measured through the ability to perform ADL and IADL tasks with caregiver assistance and the presence of locomotor disability which necessitated the procurement of aids to overcome the disability.

#### Statistical Analysis:

Descriptive statistics were used to present the distribution of socio-demographic characteristics, socio-economic characteristics, financial status in household and health characteristics by older adults’ living arrangements. Chi square tests were employed to identify group differences in proportions and one-way ANOVA F-tests for continuous variables. These tests were used to identify group differences in health, locomotor and functional needs by living arrangements as well as to identify associations and differences in caregiving patterns. The dependent variables were dichotomised and binary logistic regression was employed to view the adjusted effects of living arrangements on caregiving to older adults. Results are presented in the form of odds ratio (ORs) with 95% CI (confidence intervals). The estimation of confidence intervals takes into account design effects. In order to maintain representativeness of the sample and avoid oversampling of certain categories among variables, appropriate weights have been used. A *p* ≤ 0.001 was considered statistically significant. The data were analysed using the Statistical Package for the Social Sciences SPSS Inc. Released 2009. PASW Statistics for Windows, Version 18.0. Chicago: SPSS Inc.

## Results

The descriptive characteristics (Socio-Demographic, Health, Socio-Economic and Financial status in Household) of the study sample by Living Arrangements are depicted in Table 1. The 9850 respondents were classified based on their living arrangements into 3 categories: older adults living alone/living with servant (6.2 %), those living with spouse/living with spouse and servant (14.9 %) and those older adults living with children and others (78.9 %) respectively. The proportion of older adult women who lived alone was nearly four times in comparison to older adult men. Significantly, nearly twice the number of older adult men lived with their spouses as compared to women older adults. A higher proportion of older adults living alone and living with spouse exclusively were from the lowest quintile of the wealth index. A higher proportion of older adults living with spouse and those living alone were financially independent while a higher proportion of older adults who lived alone and those who lived with spouse were dependent on any family member for financial needs.

**Table 1:** Description of the study sample (socio-demographic, health and socio-economic characteristics of older adults) by their living arrangements (*N* = 9850)

	Living alone (*n* = 612; 6.2 %)	Living with spouse only (*n* = 1468; 14.9 %)	Living with children and/or others (*n* = 7770; 78.9 %)	Total *N* = 9850	*p-*value
Socio-demographic characteristics					
Gender					
Men	2.5	20.1	77.4	4672	<0.001
Women	9.6	10.2	80.2	5178	
Residence					
Rural	6.5	17.3	76.0	5137	<0.001
Urban	5.8	12.2	81.9	4713	
Age group					
60–69 years	6.0	15.7	78.3	6238	<0.001
70–79 years	6.9	15.2	78.0	2600	
80 + years	5.9	9.4	84.7	1012	
Marital status					
Currently married/living together	0.7	24.8	74.5	5885	<0.001
Currently single	14.4	0.3	85.4	3965	
Children					
Mean number of children (Mean ± SD)	2.71 ± 1.83	3.02 ± 1.67	3.46 ± 1.80	3.35 ± 1.80	
Mean number of sons (Mean ± SD)	1.30 ± 1.27	1.44 ± 1.22	1.87 ± 1.16	1.78 ± 1.19	
Mean number of daughters (Mean ± SD)	1.42 ± 1.23	1.58 ± 1.23	1.59 ± 1.34	1.58 ± 1.32	
Socio-economic characteristics					
Wealth index (*n* = 9844)					<0.001
Lowest quintile (Q1)	15.5	22.7	61.8	1954	
Second quintile (Q2)	7.3	16.1	76.5	1974	
Middle quintile (Q3)	5.0	13.6	81.4	1938	
Fourth quintile (Q4)	1.8	11.0	87.2	1961	
Highest quintile (Q5)	1.6	11.2	87.2	2017	
Financial status in household					
Not dependent	11.7	24.7	63.5	2488	<0.001
Partially dependent	4.8	12.1	83.2	2431	
Fully dependent	7.9	10.6	81.5	659	
Financial dependence on (*n* = 4993)					
Spouse	0.01	29.4	69.6	2241	<0.001
Any Family	16.8	34.5	48.5	1912	
Other relatives/friends/NGO, community, etc.	0.2	14.2	65.7	840	

*p-value* is from chi square tests for categorical variables

Table 2 represents the distribution and differences in health and functional status by living arrangements of older adults. A higher proportion of older adults living with spouse rated their general health status as excellent/very good compared to the rest. A higher proportion of older adults who lived alone and those who lived with children and all others had suffered from an acute sickness episode in the past 15 days as compared to older adults who lived with their spouse/partner. A higher proportion of older adults who lived with children and others had suffered from a hospitalization in the past 1 year, suffered from the presence of any chronic morbidity, reported of any locomotor disability (vision, hearing, walking, chewing, speaking and memory) and had higher ADL limitations as compared to older adults living with spouse/partner and those living alone. Older adults living with children and others also had higher mean ADL limitations score (2.87 ± 1.83) as compared to older adults living with spouse/partner (2.50 ± 1.76) and older adults living alone (1.75 ± 1.63). Only with respect to IADL limitations did older adults living with children and others have better outcomes.

**Table 2:** Distribution and differences in health and functional status by living arrangements

	Living alone (*n* = 612; 6.2 %)	Living with Spouse Only (*n* = 1468; 14.9 %)	Living with spouse, children and others (*n* = 7770; 78.9 %)	Total	*p-*value
Health and functional characteristics					
General health status (*n* = 9830)					
Excellent/Very Good	3.6	16.3	80.1	1604	<0.001
Good/Fair	6.7	15.5	77.7	6538	
Poor	6.6	11.3	82.1	1688	
Acute sickness score (*n* = 1227)					
No ailment	6.2	15.2	78.6	8621	
Any ailment	6.6	12.7	80.7	1227	
Hospitalization score (*n* = 923)					
No hospitalization	6.3	14.9	78.7	8921	0.947
Any hospitalization	5.4	14.2	80.4	929	
Chronic morbidity (*n* = 9850)					
No chronic morbidity	6.8	17.7	75.5	3494	<0.001
Any chronic morbidity	5.9	13.4	80.7	6356	
Locomotor disability score (*n* = 9850)					
No disability	6.7	19.3	74.0	2970	<0.001
Any one disability	6.0	13.0	81.0	6880	
ADL limitations					
No ADL limitations	6.3	15.5	78.2	9112	<0.001
Any ADL limitation	4.5	7.9	87.7	738	
Mean ADL limitations score (Mean ± SD)	33 (1.75 ± 1.63)	58 (2.50 ± 1.76)	647 (2.87 ± 1.83)	738 (2.79 ± 1.83)	<0.001
IADL limitations					
No IADL limitations	0	1	0	1	<0.001
Any IADL limitations	612	1467	7770	9849	
Mean IADL limitations score (Mean ± SD)	5.97 ± 2.05	5.60 ± 1.84	4.78 ± 2.30	4.97 ± 2.26	<0.001

*p*-value is from chi square tests for categorical variables and one-way ANOVA *F*-tests for continuous variables

Caregiving can be very diverse in nature with the commonly noticed tangible acts being instrumental caregiving and financial support. The distribution and differences in caregiving (instrumental) to older adults for hospitalization and ADL limitations by living arrangements are presented in Table 3. During older adults’ hospitalization episode, caregivers who stayed with the older adult in the hospital were considered. In case of ADL limitations, instrumental assistance in dealing with ADL limitations was considered. Among older adults living alone, a higher proportion of them had no one to support them during times of hospitalization. A higher proportion of older adults living with spouse/partner obtained care from their spouse and older adults living with children were most commonly cared for by the family. For ADL limitations, the spouse was the commonest caregiver for older adults living with spouse while the family cared for a higher proportion of older adults living with children and others. Older adults living alone had very little spousal and family contribution for ADL tasks and a higher proportion of them also received care from non-family sources.

**Table 3:** Distribution and differences in caregiving by living arrangement and type of health and functional needs

	Living alone (*n* = 612; 6.2 %)	Living with spouse (*n* = 1468; 14.9 %)	Living with spouse, children and others (*n* = 7770; 78.9 %)	Total	*p-*value
Caregivers					
Caregiver during hospitalization (who stayed with older adult; *n* = 929)					
No One (self)	27.0	18.9	54.1	37	<0.001
Spouse	0.7	26.0	73.3	277	
Family	6.8	7.7	85.4	542	
Non-Family	1.4	15.1	83.6	73	
Main caregiver for ADL limitations (*n* = 719)					
Spouse	0.7	30.7	68.7	150	<0.001
Family	2.8	1.8	95.4	505	
Non-Family	20.3	4.7	75.0	64	

All *p* values are chi-square *p* values

Next, we looked at caregiving through financially supporting older adults for their treatment expenses. Table 4 presents the distribution and differences by caregivers who paid for treatment needs of older adults. For acute sickness, non-family contribution towards the medical expenses of older adults living alone was high followed by self-financed care. In case of older adults living with spouse, spousal contribution was highest in proportion followed by self-financed care. For older adults living with children and others, the family was the primary support financially. Chronic morbidity expenses of older adults who lived alone were mainly handled by non-family sources of caregiving. The primary caregiver for older adults living with spouse was the spouse and it was the family which majorly supported the chronic morbidity expenses of older adults living with children and others. For provision of aids to overcome locomotor disability, older adults who lived alone were mainly supported by voluntary agents and others while older adults living with spouse were financially supported mainly by others. Children were the main financial support for older adults who lived with children and others.

**Table 4:** Distribution and differences in caregivers who paid for the treatment needs of older adults by living arrangements

	Living alone (*n* = 612; 6.2 %)	Living with spouse (*n* = 1468; 14.9 %)	Living with spouse, children and others (*n* = 7770; 78.9 %)	Total	*p-*value
Health characteristics					
Caregiver for acute sickness (who paid) (*n* = 955)					
No one (self)	7.7	16.1	76.2	311	<0.001
Spouse	6.3	33.6	60.2	128	
Family	4.6	5.7	89.8	459	
Non-Family	17.5	7.0	75.4	57	
Caregiver for chronic morbidity (*n* = 4966)					
No one (self)	8.7	20.8	70.6	1848	<0.001
Spouse	2.0	28.7	69.4	356	
Family	2.7	6.2	91.1	2513	
Non-Family	12.0	6.8	81.1	249	
Caregiver for locomotor disability (*n* = 3547)					
No one (self)	6.3	15.9	77.8	2076	<0.001
Children	2.8	3.4	93.8	1204	
Voluntary Agents	8.5	8.5	83.1	59	
Others	6.7	28.8	64.5	208	

All *p* values are chi-square *p* values

Table 5 presents the unadjusted and adjusted ORs for caregiver being ‘none’ as against the caregiver being ‘any family or non-family member’ by the socio-demographic, socio-economic, and health characteristics of older adults using logistic regression models. The unadjusted ORs provide the effect of living arrangements on caregiving to older adults for hospitalization, locomotor disability, ADL limitations, chronic morbidity and acute sickness. The act of caregiving for hospitalization (who stayed with them during hospitalization) and caregiving for ADL refers to instrumental caregiving. For acute sickness, chronic morbidity and locomotor disability, the caregiving act refers to financially supporting older adults. For ADL limitations, the contribution of self (no one) caregiving was not available in the dataset; hence, the contribution of spouse versus all others has been explored in the logistic regression (not shown in table). The adjusted model includes all other independent variables.

**Table 5:** Unadjusted and adjusted effects (ORS, 95 % CIS) of living arrangements and other independent variables on caregivers for different care needs of older adults

	Caregiver for hospitalization (None Vs. Any Caregiver)		Caregiver for locomotor disability (None Vs. Any Caregiver)		Caregiver for chronic morbidity (None Vs. Any Caregiver)	
	Unadjusted (OR)	Adjusted (aOR)	Unadjusted (OR)	Adjusted (aOR)	Unadjusted (OR)	Adjusted (aOR)
Living arrangements						
Living Alone + With Servant^R^ (*n* = 612; 6.2 %)	1.000	1.000	1.000	1.000	1.000	1.000
Living with Spouse + Living with Spouse and Servant (*n* = 1468; 14.9 %)	3.785 (1.07–14.42)	2.693 (0.46–15.80)	0.487 (0.28–0.87)	1.605 (0.82–3.13)	0.988 (0.64–1.52)	2.166 (1.31–3.57)
Living with All Others (Spouse and/or Children (*n* = 7770; 78.9 %)	8.723 (2.94–25.88)	6.885 (1.73–27.44)	1.421 (0.91–2.23)	2.606 (1.54–4.41)	2.884 (1.99–4.19)	4.614 (3.01–7.07)
Socio-demographic characteristics						
Gender						
Men^R^		1.000		1.000		1.000
Women		5.838 (1.34–25.48)		3.490 (2.58–4.73)		2.801 (2.22–3.53)
RESIDENCE						
Rural^R^		1.000		1.000		1.000
Urban		0.421 (0.14–1.31)		0.682 (0.53–0.88)		0.801 (0.66–0.97)
Age group						
60–69 years^R^		1.000		1.000		1.000
70–79 years		0.955 (0.30–3.02)		1.090 (0.83–1.43)		1.169 (0.96–1.43)
80 + years		0.766 (0.17–3.51)		2.215 (1.52–3.22)		2.553 (1.87–3.48)
Marital status						
Currently Single^R^		1.000		1.000		1.000
Currently Married/Living Together		3.484 (0.74–16.37)		0.576 (0.42–0.80)		0.880 (0.68–1.13)
Children						
Mean number of children (Mean ± SD)		1.236 (0.89–1.72)		1.105 (1.03–1.19)		1.099 (1.04–1.16)
Socio-economic characteristics						
Wealth index						
Lowest quintile (Q1)^R^		1.000		1.000		1.000
Second quintile (Q2)		0.932 (0.28–3.11)		1.063 (0.65–1.74)		0.930 (0.68–1.26)
Middle quintile (Q3)		3.527 (0.61–20.38)		1.440 (0.90–2.31)		0.998 (0.74–1.35)
Fourth quintile (Q4)		9.158 (0.91–91.99)		0.889 (0.554–1.43)		0.960 (0.70–1.31)
Highest quintile (Q5)		5.908 (0.91–38.38)		0.605 (0.37–0.98)		0.631 (0.46–0.87)
Financial status in household						
Not dependent^R^		1.000		1.000		1.000
Partially dependent		0.531 (0.16–1.79)		2.218 (1.66–2.95)		2.324 (1.90–2.85)
Fully dependent		0.329 (0.07–1.62)		2.709 (1.83–4.00)		3.887 (2.92–5.19)
Health characteristics						
General health status						
Excellent/V. Good^R^		1.000		1.000		1.000
Good/Fair		1.651 (0.43–6.28)		0.745 (0.53–1.04)		0.898 (0.70–1.16)
Poor		3.474 (0.71–17.11)		1.127 (0.76–1.68)		1.103 (0.82–1.49)

*OR* odds ratio, *aOR* adjusted odds ratio, *R* reference category

### Effects of Living Arrangements on Caregiving to Older Adults for Their Care Needs

Considering caregivers for hospitalization, the unadjusted model shows that older adults living with spouse were more than 3 times likely to obtain care from ‘any family or non-family caregiver’ (OR 3.785; CI 1.07–14.42) and those older adults living with children and all others were more than 8 times more likely to receive support from ‘any caregiver’ (OR 8.723; CI 2.94–25.88) as compared to older adults living alone (reference category). This has remained significant in the adjusted model. In case of caregivers for locomotor disability, older adults living with spouse were less likely to obtain care from ‘any caregiver’ (OR 0.487; 0.28–0.87) as compared to the other categories. In the adjusted model, this likelihood altered with older adults living with spouse being more likely to receive care from ‘any caregiver’ (aOR 1.605; CI 0.82–3.13) and older adults living with children and all others 2.6 times more likely to receive care from ‘any caregiver’ (aOR 2.606; 1.54–4.41) as compared to reference category. For ADL limitations (not shown in table), the unadjusted model showed that older adults living with spouse were more likely to receive care from the spouse (OR 15.267; CI 3.47–67.13). For chronic morbidity needs, the unadjusted model showed that older adults living with spouse were less likely to receive care from ‘any caregiver’ (OR 0.988; CI 0.64–1.52) while older adults living with children and others were nearly three times more likely to receive care from ‘any caregiver’ (OR 2.884; CI 1.99–4.19). In the adjusted model controlling for other variables, older adults living with spouse were 2 times more likely to obtain care from any caregiver (aOR 2.166; CI 1.31–3.57) while older adults living with children and others were more than 4 times more likely to receive care from any caregiver (aOR 4.614; CI 3.01–7.07) as compared to reference category. Considering caregiver for acute sickness (not shown in table), the adjusted model showed that older adults living with spouse were nearly 1.5 times more likely to be cared for by any caregiver (OR 1.456; CI 0.57–3.73) while older adults living with children and others were 1.3 times more likely to be cared for by any caregiver for their acute sickness needs (OR 1.305; 0.59–2.87) as compared to reference category.

### Effect of Control Variables on Caregiving to Older Adults During Care Needs

The discussion of the adjusted effects of the control variables focuses on all the 5 adjusted models in Table 5. With other variables controlled for, wealth index had a significant positive effect on any caregiver being available to care for the older adult during hospitalization. Gender-wise, being a woman had a significant positive effect on having any caregiver for hospitalization (OR 5.838; CI 1.34–25.48), locomotor disability (OR 3.490; CI 2.58–4.73), chronic morbidity (OR 2.801; CI 2.22–3.53) and acute sickness (OR 1.377; CI 0.82–2.31). By place of residence, urban older adults had more likelihood of obtaining care from any caregiver in case of ADL limitations (not shown in table) (OR 1.621; CI 0.50–5.24) and acute sickness (not shown in table) (OR 1.029; CI 0.67–1.57). For older adults, advanced age (80 + years) had a significant and positive likelihood on a caregiver being available to care for older adults in case of locomotor disability (OR 2.215; CI 1.52–3.22), ADL limitations (OR 3.646; CI 1.01–13.13), chronic morbidity (OR 2.553; CI 1.87–3.48) and acute sickness (OR 1.042; CI 0.54–2.02). Being married/living together had a significant and positive likelihood of a caregiver being available to care for older adults in case of hospitalization (OR 3.484; CI 0.74–16.37) and ADL limitations (OR 10.599; CI 1.28–87.86). With respect to children being available, mean number of children had a significant and positive likelihood on a caregiver being available to care for older adults in case of hospitalization, locomotor disability, chronic morbidity and acute sickness. Considering financial status in the household, older adults who were partially or fully financially dependent on the household were more likely to have a caregiver to care for them in case of locomotor disability, ADL limitations, chronic morbidity and acute sickness.

## Discussion

This study has utilised data from a large representative sample for older adults in India. While describing the characteristics of older adults in India as represented by their gender, age group, place of residence, marital status, living arrangements, and socio-economic characteristics, the study has importantly aimed to explore how the care needs of older adults and care provision to them differ by living arrangements for different care-requiring situations. The care-requiring situations considered here include acute sickness, sickness requiring hospitalization, chronic morbidity, functional disability represented by ADL and IADL difficulties, and locomotor disability.

The paper has aimed to answer five dimensions: (1) whether care needs are different for older adults across socio-demographic, socio-economic and health characteristics and by their living arrangements, (2) whether caregiving to older adults varied for different care needs by living arrangements, (3) to estimate the contribution of spouse, family members and non-family members (relative/friend) in care provision to older adults for different care needs by their living arrangements, (4) to demonstrate effects of living arrangements on caregiving and (5) to identify care gaps that the analysis brings forth.

From the results, living arrangements were significantly associated with health and functional status as well as caregiving patterns. This study found that while living alone did not substantially disadvantage older adults with respect to ADL limitations or acute sickness or hospitalization, it was significantly associated with a higher proportion of locomotor disability, chronic morbidity and higher IADL limitations. Zyzanski et al. 1989, had reported that older adults living with children had worse outcomes as compared to those who lived alone while there were other studies who found the converse, i.e., that living alone had disadvantaged individuals with regards to functional health (Macran et al. 1996; Denton and Walters 1999) and other measures of health.

While our findings conform to those from prior research on the contribution of family, especially children and spouse apart from other family members towards general care provision for older adults in India (Cicirelli 1990; Gupta and Pillai 2002); they bring newer insights into care provision for specific care needs of older adults by their living arrangement. The contribution of relatives, friends and non-family contacts which was hitherto assumed to be less and not quantified is seen to be emerging through this sample. Non-family contribution to caregiving is accounting for around 8–10 % of the caregiving contribution across older adults’ care requirements.

This study also found that co-residence, which allows the pooling of resources and supports individual family members in times of need (Husain and Ghosh 2011), is very much existent in India and plays a crucial role in the care and support to older adults. Further, co-residence of older adults with children and significant others is likely to assure continued care and support, although smaller contributions from relatives, friends and others outside the family or household are emerging. Presence of the spouse has ensured care availability to the older adult requiring care. Messeri et al. (1993) had earlier found that an available spouse usually provides all the much needed care to an older adult requiring care; it is only when the spouse is absent that available children are likely to provide care more than other relatives or friends. The ‘any family’ member category accounts for the highest proportion among caregivers to older adults, rightly so since it includes children, sons-in-law/daughters-in-law and grand-children. This typically exemplifies the intergenerational bonding and co-residence of parents with their children and grandchildren in India which ensures care availability to the older adults. It also highlights the care gaps that could arise when older adults begin to live alone and brings to the fore the self-dependence that older adults living alone resort to even in failing health and functional status.

The study also found that older adults who lived alone had a better disability status as compared to those who lived with children and others. What this probably indicates is that living alone is in part influenced by the ability to independently handle routine tasks; however, in the Indian situation this must be read cautiously since the choice to live alone is often not the decision of the older adult and could be the result of being vulnerable and socially rejected by family and others. On the one hand, living alone might mean financial ability to live independently, while on the other hand it might also point to social isolation and social deprivation from one’s family (Tohme et al. 2011) which might be closer to reality in the Indian context given the low financial independence of older adults. Kodoth and Rajan (2008) had found that it was likely that older adults who lived alone were either without bequeathable assets and thus had a relatively higher likelihood of residence in old-age homes, living alone, and being looked after by relations other than their children when widowed.

One peculiarity in the findings was that even though older adults lived alone, the proportion of care and support they received from their family was still high. It is difficult to say whether older adults actually received all this care when living alone or whether they chose not to report care deficit from the family as it is a taboo to reveal such issues in India. The findings however indicate to us that there was familial help available to older adults in care-requiring situations even though they lived alone. Whether this unearths a relatively recent trend in living arrangements, appropriately referred to as “living apart but together”, wherein joint family co-residence is discontinued but strong social support is immediately available, particularly in times of health crises (Sokolovsky 2001) needs to be further explored.

Despite the relevance of these findings, we recognize the limitations in the study. This being a cross-sectional study is unable to capture the nuances that caregiving and care-receiving entail for the older adult and the longitudinal changes that arise with advancing age. The cross-sectional nature of the data also brings forth the issue of endogeneity that likely exists between caregiving and living arrangements. We could not address other parameters of older adult life such as life satisfaction, emotional care, communication needs and holistic ageing in this study. The health of both spouses was also not available in the data which would have been vital information since the nature and availability of spousal help depends on the health status of both partners (Feld et al. 2004). Another important dimension which could not be answered through this analysis is the recognition that as life expectancy of older adults increase and they remain healthy at advanced ages, older men and women are also able to contribute to the well-being of their families and communities too (Hughes et al. 2007; Verbugge and Chan 2008; Silverstein et al. 2002). This reciprocal nature of caregiving and support is often less recognized as societies begin to age. This biased thought process has resulted in older adults being labelled as a worrisome burden in rapidly evolving societies such as in India. It would be interesting to qualitatively explore who the actual caregivers among the family and non-family sources are and how the exchange of care between older adults and their significant others ensues in the household setting (Dowd 1980).

In conclusion, this study advances knowledge about caregiving to older adults in India for health, functional and disability needs by their living arrangements. It reinforces that informal care to older adults is the mainstay in the Indian context and brings forth the care gaps that exist for older adults living alone. It also acknowledges that non-family sources of caregiving are steadily becoming visible, especially for those older adults living alone or away from their spouse and children. Although family-based caregiving is still the norm for older adults, the fact that there is a steady increase in older adults living alone or living away from the traditional joint family is going to challenge care availability and provision to them. Increasing nuclearization of families and migration of children away from the traditional multi-generational households are important areas of concern when we think of the care needs of older adults. Significant directions can be found for research and policy from this study on how living arrangements of older adults dictate care provision to them and how older adults might continue to benefit from family-based caregiving as hitherto formal caregiving and assisted living is near negligible in the Indian context. A deeper understanding of the process by which families and who within the family living arrangement comes to assume the responsibility of caring for the older adult is very essential for planning, designing and evaluation of older adult support policies. As the Indian Government encourages family-based caregiving and co-residence for the benefit of the elderly, initiatives such as tax incentives for a co-residing adult child might be a model for ensuring care availability for older adults within the household (Government of India (GOI) 2007). Care gaps for older adults living alone have come to the fore and concerted efforts are necessary to ensure that those older adults who have substantial care needs are not disadvantaged on account of their living arrangement.
